# Development of Shrimp Freshness Indicating Films by Embedding Anthocyanins-Rich *Rhododendron simsii* Flower Extract in Locust Bean Gum/Polyvinyl Alcohol Matrix

**DOI:** 10.3390/ma15217557

**Published:** 2022-10-27

**Authors:** Chenchen Li, Dawei Yun, Zeyu Wang, Fengfeng Xu, Chao Tang, Jun Liu

**Affiliations:** College of Food Science and Engineering, Yangzhou University, Yangzhou 225127, China

**Keywords:** *Rhododendron simsii*, flower anthocyanins, locust bean gum, freshness indicating, smart packaging, antioxidant activity, shrimp

## Abstract

Freshness indicating films containing anthocyanins are one type of smart packaging technology. Anthocyanins in the films can show visual color changes when food spoilage occurs, thereby indicating the freshness degree of food in real-time. *Rhododendron simsii* is a landscape plant with attractive flowers that are abundant in anthocyanins. In this study, smart packaging films were prepared by embedding 2% and 4% *R. simsii* flower anthocyanins (RA) in locust bean gum- (LBG) and polyvinyl alcohol- (PVA) based matrices. The micro-structure, barrier, mechanical, thermal, antioxidant, and color-changeable properties of the films were determined. The potential application of the films in indicating the freshness of shrimp at 4 °C was also investigated. Results showed that the RA interacted with the LBG/PVA matrices through hydrogen bonds, which significantly improved the barrier, mechanical, thermal, antioxidant, pH-sensitive, and ammonia-sensitive properties of the films. Meanwhile, the performance of the films was remarkably influenced by the content of the RA. The film containing 4% RA had the highest light blocking ability, tensile strength (38.32 MPa), elongation at break (58.18%), and antioxidant activity, and also showed the lowest water vapor permeability (22.10 × 10^−11^ g m^−1^ s^−1^ Pa^−1^) and oxygen permeability (0.36 cm^3^ mm m^−2^ day^−1^ atm^−1^). The films containing 2% and 4% RA could effectively change their colors when the level of total volatile basic nitrogen in the shrimp exceeded the safe value, which demonstrated the suitability of the films for indicating the freshness degree of shrimp.

## 1. Introduction

To meet the preferences and expectations of consumers in food packaging, smart packaging technology has attracted extensive attention in recent years [1]. In contrast to traditional packaging, smart packaging technology can supply visual information on the storage condition and freshness degree of food [2,3]. Freshness indicating films are one type of smart packaging technology, and are normally developed by embedding natural pigments within biopolymer-based matrices [4]. The principle of freshness indicating films is based on the release of volatile ammonia compounds during the spoilage of protein-rich foods (e.g., meat and aquatic products), which can be sensed by the color-changeable natural pigments in the films. Freshness indicating films are able to inform the freshness degree of protein-rich foods through the color changes of natural pigments [5,6]. To date, different types of natural pigments, including anthocyanins, chlorophyll, and curcumin, have been embedded in polysaccharide-/protein-based matrices to produce freshness indicating films [7,8]. Among them, anthocyanins are frequently selected for the production of freshness indicating films, because anthocyanins have wide distributions in plants (e.g., vegetables, fruits, grains and flowers) and possess excellent antioxidant and color-changeable properties [9,10]. Meanwhile, anthocyanins are safe and edible, while showing beneficial health effects [11]. Till now, studies have revealed that the source of the anthocyanins [12], the nature of the biopolymer, [13] and the storage condition of the films [14] are important factors affecting the functionality of freshness indicating films. It has been demonstrated that anthocyanins from flowers, including butterfly pea [15], rose, [16] and roselle [17], can be used to develop freshness indicating films. However, the development of freshness indicating films based on the anthocyanins from other flowers is very limited.

*Rhododendron simsii* Planch., a landscape plant with attractive flowers, is wildly distributed in China, Japan, Laos, Myanmar and Thailand [18]. *R. simsii* generally blooms in spring and presents diverse flower colors, such as white, red, carmine red, pink, purple, and lilac [19]. *R. simsii* flowers have been used as a traditional Chinese medicine for the treatment of bronchitis, pain, and coronary heart disease for thousands of years [20]. The pharmacological effects of *R. simsii* flowers are mainly attributed to their polyphenolic compounds with potent antioxidant, anti-inflammatory, and antifibrotic activity [21]. Meanwhile, anthocyanins are responsible for the attractive colors of *R. simsii* flowers [22]. Studies have demonstrated that cyanidin glycosides were the major anthocyanins in *R. simsii* flowers [22,23]. Considering the high content of anthocyanins in *R. simsii* flowers, it is supposed that *R. simsii* flower anthocyanins (RA) could be used to prepare freshness indicating films. Nonetheless, no studies have been conducted to prepare freshness indicating films using RA.

Here, we developed freshness indicating films by embedding RA in locust bean gum- (LBG) and polyvinyl alcohol- (PVA) based matrices. The selection of LBG/PVA as the matrix was because neutral polysaccharides were beneficial for the stabilization of the anthocyanins [13]. Based on our preliminary study, the films containing 2% and 4% RA had ideal colors. In this study, the structural characterization, physical properties, and functional properties of films with different RA contents (0, 2% and 4%) were compared. In addition, the potential application of the films in indicating the freshness of shrimp at 4 °C was investigated.

## 2. Materials and Methods

### 2.1. Material and Reagents

Fresh *R. simsii* flowers were collected from the campus of Yangzhou University (Yangzhou, China) on May 2020. Macroporous resin AB-8 (particle size: 0.3−1.25 mm and average pore size: 13−14 nm), used for the purification of the anthocyanins, was purchased from the Donghong Chemical Co. (Zibo, China). Ethanol, hydrochloric acid (HCl), Folin–C iocalteau reagent, LBG (molecular weight: 310 kDa), PVA (degree of polymerization: ~1750), glycerol, and 2,2-diphenyl-1-pyrrolhydrazino (DPPH) were purchased from the Macklin Biochemical Co. (Shanghai, China).

### 2.2. Extraction and Purification of Anthocyanins

Fresh *R. simsii* flowers were freeze-dried, pulverized and extracted with 80% ethanol containing 0.5% HCl at 4 °C for 10 h. The residue was repeatedly extracted two more times. The extract solution was combined and centrifuged at 8000 rpm for 15 min, and the supernatant was collected and concentrated by a rotary evaporator at 45 °C. The concentrate was loaded on an AB-8 resin column (50 cm × 2.6 cm), which was first eluted with distilled water to remove impurities, and then eluted with 25% ethanol containing 0.5% HCl [24]. The eluate was collected, dried under vacuum, and named as RA. The total anthocyanin content of the RA was determined as 111.38 mg cyanidin-3-O-glucoside equivalent per g of dried extract by the pH differential method [25].

### 2.3. Preparation of Films

The film preparation method referred to Wu et al. [26], with some modifications. First, 170 mL of a LBG/PVA blend matrix solution containing 2% LBG and 1% PVA was prepared at 95 °C for 4 h. After the matrix solution was cooled to 40 °C, 2% (0.102 g) or 4% (0.204 g) of the RA, based on the total mass of the LBG and PVA, was added into the matrix solution. After that, 20% (1.02 g) of the glycerol, based on the total mass of LBG and PVA, was dropwisely added to the solution and stirred at room temperature for 30 min. The obtained film-forming solution was degassed, poured onto a Plexiglas plate (24 cm × 24 cm), and dried at 30 °C for 48 h. LBG/PVA films containing 0, 2%, and 4% of the RA were named as LP, LPRA-2, and LPRA-4 films, respectively. All the films were equilibrated at 25 °C and 50% relative humidity (RH) for 48 h.

### 2.4. Structural Characterization of Films

For micro-structural determination, the film samples were first fractured in liquid nitrogen, and then a cross-section of each film sample was observed using GeminiSEM 300 scanning electron microscopy (Carl Zeiss, Oberkochen, Germany) at 2 kV. For Fourier transform infrared (FT-IR) spectrum determination, each film sample was recorded by a Varian 670 spectrometer (Varian, Palo Alto, CA, USA) in attenuated total reflection mode with a wavenumber range of 400−4000 cm^−1^ [27].

### 2.5. Determination of Physical and Functional Properties of Films

#### 2.5.1. Thickness

The thickness of each film at 10 locations was measured using an EVERTE digital micrometer (Bonthe Corp., Shangqiu, China).

#### 2.5.2. Color

The color parameters of each film were measured using a SC-80C colorimeter (Kangguang Corp., Beijing, China), while a photo of each film was captured with a camera. Three replications were performed for each kind of film.

#### 2.5.3. Light Transmittance

The light transmittance of each film in the wavelength of 200−800 nm was measured using a 759S UV/Vis spectrophotometer (Lengguang Corp., Shanghai, China). Three replications were performed for each kind of film.

#### 2.5.4. Water Vapor Permeability (WVP)

Each film sample (6 cm × 6 cm) was tightly covered on a centrifuge tube filled with 40 g of silica gel. The tube was placed in a desiccator with 100 mL of distilled water at 25 °C, and the tube was weighed every 24 h for 7 days [28]. Three replications were performed for each kind of film.

#### 2.5.5. Oxygen Permeability (OP)

Each film sample (10 cm in diameter) was placed between two chambers of a Basic 201 gas permeability tester (Labthink Corp., Jinan, China) at 23 °C and 50% RH [29]. Air in the two chambers was thoroughly evacuated, and then oxygen gas was inflated into the upper chamber. The pressure change in the bottom chamber was recorded as a function of time. Three replications were performed for each kind of film.

#### 2.5.6. Water Contact Angle (WCA)

To measure the WCA of the film, 2 μL of distilled water was dropped on the surface of each film. The image of the water droplet was recorded in 5 s by a GP-50 horizontally installed HD video microscope equipped with a charge-coupled device camera (Gaopin Corp., Suzhou, China) under an LED cold light source. Five replications were performed for each kind of film. The images were analyzed for contact angles using ImageJ software [30].

#### 2.5.7. Mechanical Properties

Each film sample (6 cm × 1 cm) was fixed between two clamps with an initial spacing of 4 cm, and then stretched at a rate of 6 cm/min until the film fractured [31]. The tensile strength and elongation at break of each film were recorded by a STX200 testing machine (Yishite Corp., Xiamen, China). Six replications were performed for each kind of film.

#### 2.5.8. Thermogravimetric Analysis (TGA)

Each film sample (2 mg) was heated from room temperature to 700 °C by an HTG-1 analyzer (Henven Corp., Beijing, China) under high-purity nitrogen gas with a flow rate of 20 mL/min [24].

#### 2.5.9. Antioxidant Activity

Each film sample (1.5 cm × 1.5 cm) was placed in 5 mL of distilled water, 50% ethanol, and 95% ethanol, respectively, at room temperature for 1 h. Then, the total phenol content and antioxidant activity released from the film were determined according to the method from Sani et al. [32]. Briefly, 1 mL of film sample solution was thoroughly mixed with 1 mL of 10-times-diluted Folin–Ciocalteu reagent in the dark for 5 min, which was followed by the addition of 5 mL of saturated sodium carbonate solution to react at room temperature for 2 h. The total phenol content was determined by measuring the solution absorbance at 760 nm. Meanwhile, another 1 mL of film sample solution was thoroughly mixed with 4 mL of 0.1 mmol/L DPPH methanol solution to react at room temperature in the dark for 1 h. The DPPH scavenging activity was determined by measuring the solution absorbance at 517 nm. Three replications were performed for each kind of film.

#### 2.5.10. pH-Sensitivity and Ammonia-Sensitivity

The pH sensitivity of the RA was determined by dissolving 1 mg of RA in 3 mL of different buffers (pH 3−12) for 2 min. Then, the color and 450−700 nm visible spectrum of the RA solution were recorded. The pH sensitivity of each film sample (1 cm × 1 cm) was determined in 50 mL of pH 3−12 buffers for 2 min. The ammonia sensitivity of each film sample (1 cm × 1 cm) was determined in ammonia gas (1 mol/L ammonia solution) for 10−90 min [24].

### 2.6. Application of Films

Fresh shrimp (about 35 g) were placed on a Petri dish (9 cm in diameter). Then, each film sample (2 cm × 1 cm) was stuck to the inner top of the Petri dish. The Petri dish was sealed and the shrimp were stored at 4 °C for 6 days. The color of the film sample and the total volatile basic nitrogen (TVB-N) level of the shrimp were measured every 24 h [24]. Three replications were performed for each kind of film.

### 2.7. Statistical Analysis

One-way analysis of variance was performed using Duncan’s multiple comparisons method. SPSS 23.0 software (SPSS Inc., Chicago, IL, USA) was used for the statistical analysis of the data, with *p* < 0.05 set as the significant level.

## 3. Results and Discussion

### 3.1. Cross-Sectional Microstructure

**Figure 1:**
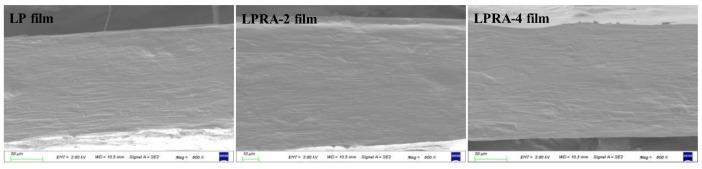
Cross-sectional micrographs of LP film, LPRA-2 film and LPRA-4 film.

The cross-sections of the LP, LPRA-2, and LPRA-4 films are shown in Figure 1. The blank LP film showed a nodular cross-sectional morphology without any cracks or holes, which indicated that the LBG and PVA had a limited compatibility, and their blend had phrase separation. Wu et al. [26] and Yao et al. [31] also reported similar results, and explained that the nodular cross-section of LP film was caused by the aggregation of LBG with high viscosity. Notably, the RA did not significantly alter the cross-sectional microstructure of the LP film, which suggests that the 2% and 4% RA were well mixed with the LBG/PVA matrices. In addition, the RA embedded in the matrix could form interactions with other film components, which uniformly distributed the RA in the film. Recently, Yao et al. [31] also found that the cross-sectional microstructure of LP film was unchanged by the natural pigment of pitaya betacyanins. However, Yun et al. [24] documented that the nodular cross-section of LP film was remarkably altered by *Loropetalum chinense* flower anthocyanins. The results indicated that the microstructure of the films was related to the source of pigment.

### 3.2. FT-IR Spectra

**Figure 2:**
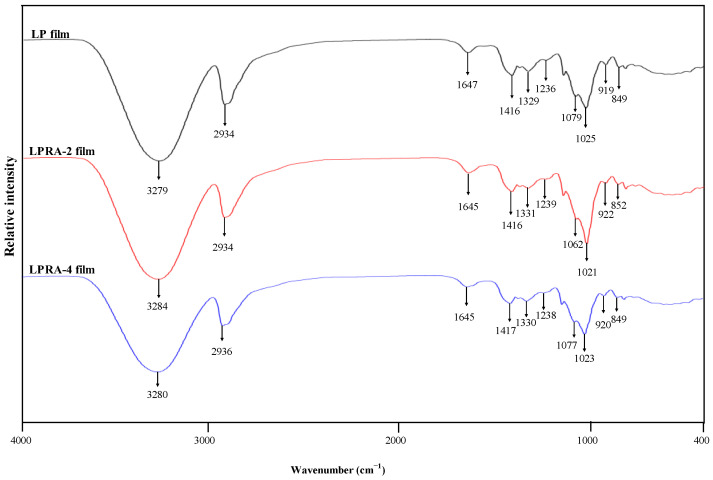
FT-IR spectra of LP film, LPRA-2 film, and LPRA-4 film.

Figure 2 shows the FT-IR spectra of the LP, LPRA-2, and LPRA-4 films. The blank LP film exhibited a broad band around 3279 cm^−1^, owing to the strong stretching vibration of hydroxyl groups (O−H) present in LBG and PVA [33]. The band around 2934 cm^−1^ corresponded to the stretching vibration of C−H methyl groups [34]. The bands at 1647 cm^−1^ and 1416 cm^−1^ were related to bound water and CH_2_ bending vibration, respectively [35]. The bands at 1025 and 1079 cm^−1^ were related to the C−O stretching vibration of the pyranose ring in the LBG [36]. After the addition of the RA, the O−H stretching band of the LP film moved to slightly higher wavenumbers (3279 and 3280 cm^−1^). In contrast, the bound water band of the LP film moved to a slightly lower wavenumber (1645 cm^−1^). The minor changes in the IR spectra were caused by the formation of hydrogen bonds between the RA and other film components, such as the LBG and PVA. Yun et al. [24] and Wu et al. [26] also found the O−H stretching band of LP film shifted after adding *L. chinense* flower anthocyanins and cockscomb flower betacyanins.

### 3.3. Thickness

The thickness of the LP, LPRA-2 and LPRA-4 films is shown in Table 1. The thickness of the LP film decreased slightly after the 4% RA was added. However, the thickness of the LP film was not significantly changed by the 2% RA. The results indicated that the incorporation of a high content (i.e., 4%) of RA could make the film become compact, which was because the RA was evenly distributed in the LBG/PVA matrix and formed interactions with other film components. Recently, Yun et al. [24] also found the thickness of LP film was decreased by 2% *L. chinense* flower anthocyanins. However, Yao et al. [31] and Wu et al. [26] documented the thickness of LP film was not significantly changed by 1% pitaya betacyanins or 4−8% cockscomb flower betacyanins.

### 3.4. Color

The appearance of the LP, LPRA-2, and LPRA-4 films is shown in Table 1. The LP film was colorless, while the LPRA-2 and LPRA-4 films were reddish brown. Meanwhile, the LPRA-4 film with a higher RA content was darker than the LPRA-2 film. The incorporation of RA greatly changed the color parameters of the LP film (Table 1). Compared with the LP film, the films with RA had lower L* values and higher Δ*E* values. The a* and b* values of the LP film were increased by the RA. The results indicated that the color of the LP film was affected by the content of anthocyanins. The LPRA-4 film with a higher RA content had a lower L* value, but higher a*, b*, and Δ*E* values. Other researchers also found that the color of LP film was changed by *L. chinense* flower anthocyanins [24], pitaya betacyanins [31], and cockscomb flower betacyanins [26].

### 3.5. Light Transmittance

**Figure 3:**
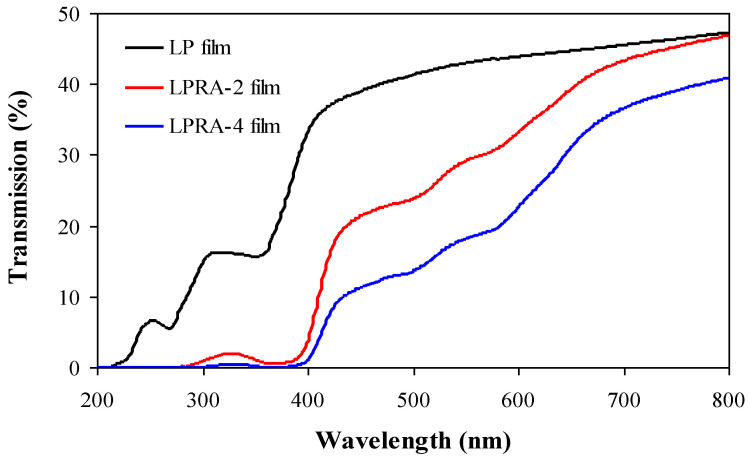
Light transmittance of LP film, LPRA-2 film, and LPRA-4 film.

Light transmittance reflects the light barrier properties of the films. Food packaging films with UV/Vis light barrier properties can effectively prevent the packaged food from absorbing light radiation [37]. As shown in Figure 3, the 2% and 4% RA greatly reduced the light transmittance of the LP film, indicating that the LPRA-2 and LPRA-4 films had good UV/Vis light barrier performance. At the same time, the light transmittance of the LPRA-4 film was lower than that of the LPRA-2 film, which was because the LPRA-4 film had a higher content of anthocyanins, and could absorb more UV/Vis light radiation [38]. Other researchers also found the light transmittance of LP film was reduced by *L. chinense* flower anthocyanins [24], pitaya betacyanins [31], and cockscomb flower betacyanins [26]. Notably, the light transmittance between 200−400 nm of the LPRA-2 and LPRA-4 films was lower than 5%, suggesting these two films could effectively block UV light-induced deterioration in food. As found in the literature, the LPRA-2 and LPRA-4 films showed similar UV/Vis light barrier properties as LP films containing *L. chinense* flower anthocyanins [24], but higher UV/Vis light barrier properties than LP films containing pitaya betacyanins [31] and cockscomb flower betacyanins [26].

### 3.6. WVP

**Figure 4:**
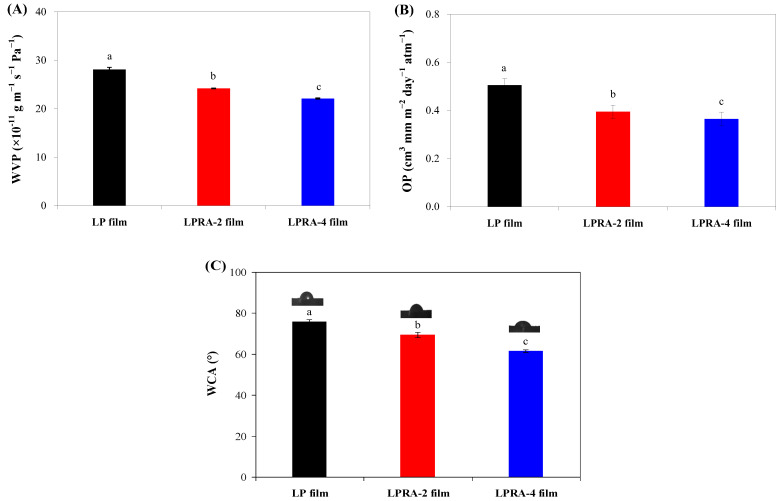
WVP (**A**), OP (**B**) and WCA (**C**) of LP film, LPRA-2 film, and LPRA-4 film. Values are given as mean ± standard deviation (n = 3 for WVP, OP, and WCA). Different lower case letters indicate the statistically significant difference (*p* < 0.05) within different films.

WVP is an index to measure how easily water vapor passes through the films. Figure 4A shows the WVP of the LP, LPRA-2, and LPRA-4 films. The WVP of the LP films decreased after adding RA. In addition, the LPRA-4 film showed a lower WVP than the LPRA-2 film. The results showed that the RA increased the water vapor barrier ability of the LP film, which was partially because RA were embedded in the film matrix and increased the compactness of the film (Table 1). Meanwhile, the RA could form inter-molecular hydrogen bonds with the hydrophilic LBG/PVA matrices, and thus reduced the affinity of the films to the water vapor [26]. Similarly, Yun et al. [24] and Wu et al. [26] found that the WVP of LP film was reduced by *L. chinense* flower anthocyanins and cockscomb flower betacyanins. However, Yao et al. [31] reported that the WVP of LP film was not remarkably changed by pitaya betacyanins. As found in the literature, the LPRA-2 and LPRA-4 films showed similar WVPs to LP films containing *L. chinense* flower anthocyanins [24] and cockscomb flower betacyanins [26].

### 3.7. OP

As shown in Figure 4B, the RA significantly decreased the OP of the LP film. With the increase in RA content, the OP of the films decreased significantly. The OP showed a similar trend to the WVP (Figure 4A), which was because the RA formed interactions with the LBG/PVA matrices and made the films become compact. In addition, anthocyanins were reported to have oxygen scavenging ability [39], which could contribute to the oxygen barrier ability of the LPRA-2 and LPRA-4 films. Recently, Yun et al. [24] also found the OP of LP film was reduced by *L. chinense* flower anthocyanins. As compared with LP film containing *L. chinense* flower anthocyanins [24], the LPRA-2 and LPRA-4 films showed lower OP. This indicated that the LPRA-2 and LPRA-4 films had good oxygen barrier abilities.

### 3.8. WCA

The hydrophilicity of the films can be analyzed by the WCA on the film surface. Figure 4C shows the WCA of the LP, LPRA-2, and LPRA-4 films. The WCA of the LP film decreased significantly with the increase in the RA content from 2% to 4%, indicating that the films gradually became more hydrophilic. This was mainly because the anthocyanins were water soluble pigments that were highly hydrophilic. Chen et al. [40] also found that the WCA of the film decreased significantly after the incorporation of purple sweet potato anthocyanins. However, in the study by Gasti et al. [41], the authors found that the low content of *Phyllanthus reticulatus* anthocyanins led to the decrease of WCA, while the high content of *P. reticulatus* anthocyanins led to the increase of WCA. Therefore, the hydrophilicity of the films was influenced by the source and the content of anthocyanins.

### 3.9. Mechanical Properties

**Figure 5:**
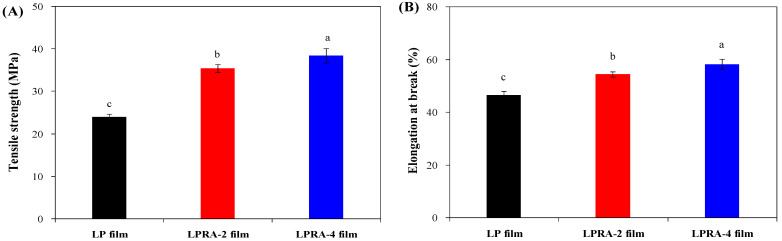
Tensile strength (**A**) and elongation at break (**B**) of LP film, LPRA-2 film, and LPRA-4 film. Values are given as mean ± standard deviation (n = 6 for tensile strength and elongation at break). Different lower case letters indicate the statistically significant difference (*p* < 0.05) within different films.

The mechanical properties, including tensile strength and elongation at break, of the LP, LPRA-2, and LPRA-4 films are shown in Figure 5. The tensile strength and elongation at break of the LP increased with the addition of the RA from 2% to 4%. The increase in TS could be illustrated by the formation of hydrogen bonds between the RA and the LBG/PVA matrix. At the same time, the RA had some plasticizing effect on the film. As a result, the stiffness and flexibility of the films were significantly increased by the RA. Similarly, Wu et al. [26] also reported that the tensile strength and elongation at break of LP film was increased by cockscomb flower betacyanins. Recently, Yun et al. [24] found the tensile strength of LP film was slightly increased by *L. chinense* flower anthocyanins, while the elongation at break of the film was somewhat decreased by anthocyanins. As found in the literature, the LPRA-2 and LPRA-4 films showed higher tensile strength than the LP films containing *L. chinense* flower anthocyanins [24] and cockscomb flower betacyanins [26].

### 3.10. Thermal Stability

**Figure 6:**
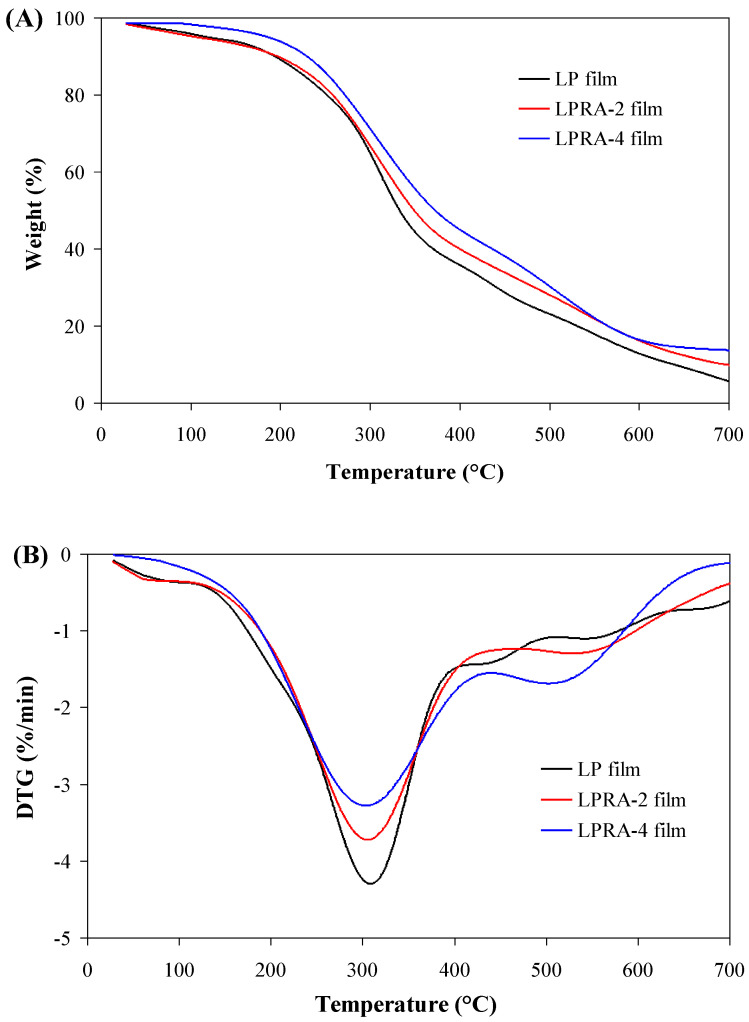
TGA (**A**) and DTG (**B**) curves of LP film, LPRA-2 film, and LPRA-4 film.

Figure 6 shows the TGA and DTG curves of the LP, LPRA-2, and LPRA-4 films. There were three stages of thermal weight loss. The first weight loss stage (room temperature to 130 °C) was dominated by water loss. The second weight loss stage was observed between 130 and 450 °C, and was attributed to the degradation of the RA, glycerol, and polymeric chains [42]. The DTG curve showed the maximum weight loss rate of the films occurred at this stage, appearing around 305 °C. The third weight loss stage was at 450–700 °C, which was due to the decomposition of the polysaccharides [43]. Notably, the LPRA-4 film degraded more slowly than the LP and LPRA-2 films, which was consistent with the relatively higher water vapor/oxygen barrier ability and mechanical properties of the LPRA-4 film. The results suggested that the LPRA-4 film had higher thermal stability than the LP and LPRA-2 films. Recently, Yun et al. [24] also found the thermal stability of LP film was increased by *L. chinense* flower anthocyanins.

### 3.11. Antioxidant Activity

**Figure 7:**
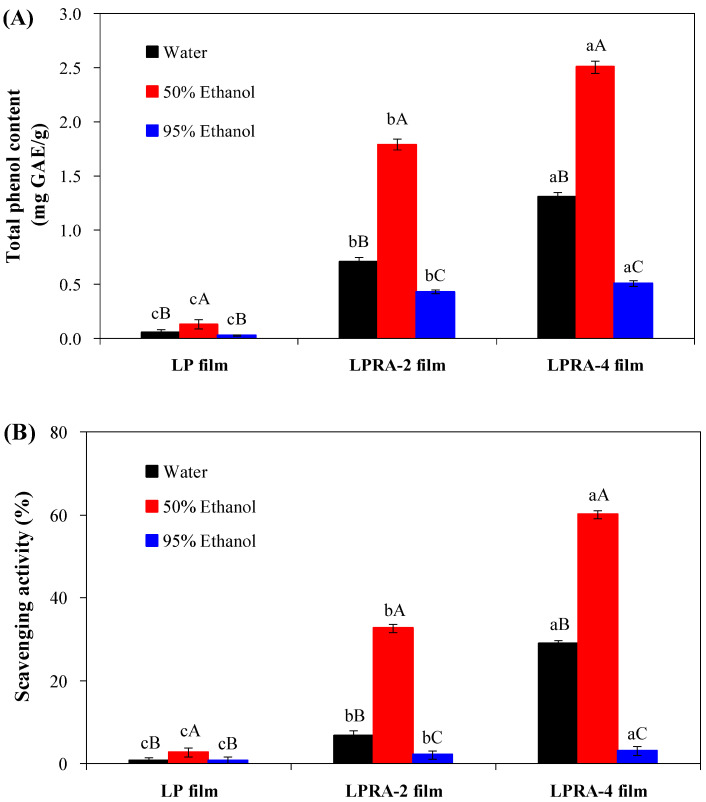
Total phenol content (**A**) and DPPH radical scavenging activity (**B**) released from LP film, LPRA-2 film, and LPRA-4 film into three different solvent systems. Values are given as mean ± standard deviation (n = 3 for total phenol content and DPPH radical scavenging activity). Different lower case letters indicate the statistically significant difference (*p* < 0.05) within different films under the same solvent. Different upper case letters indicate the statistically significant difference (*p* < 0.05) within the same film under different solvents.

The antioxidant activity of the films was tested in distilled water, 50% ethanol, and 95% ethanol, which simulated aqueous, alcoholic and fatty foods, respectively. The total phenol content released from the LP, LPRA-2, and LPRA-4 films is shown in Figure 7A. Compared with the LP film, the LPRA-2 and LPRA-4 films released higher total phenol contents in the three kinds of solvent systems, because of the presence of RA. Meanwhile, the highest total phenol content for each film was observed in 50% ethanol, followed by distilled water and 95% ethanol. This was because the RA and LBG/PVA matrices were highly hydrophilic and could swell in aqueous solutions. As a result, RA in the LPRA-2 and LPRA-4 films were more likely to be released from the film matrices into solutions. However, the hydrophilic film matrices could shrink in 95% ethanol and thus restrict the release of RA. Notably, the DPPH radical scavenging activity of the films well correlated with the total phenol content of the films, which demonstrated that the antioxidant activity of the films was mainly attributed to the RA. Recently, Yun et al. [24] also found LP film containing *L. chinense* flower anthocyanins had similar total phenol and antioxidant releasing profiles in three solvent systems. The results indicated that the LPRA-2 and LPRA-4 films had active packaging potentials.

### 3.12. pH Sensitivity and Ammonia Sensitivity

**Figure 8:**
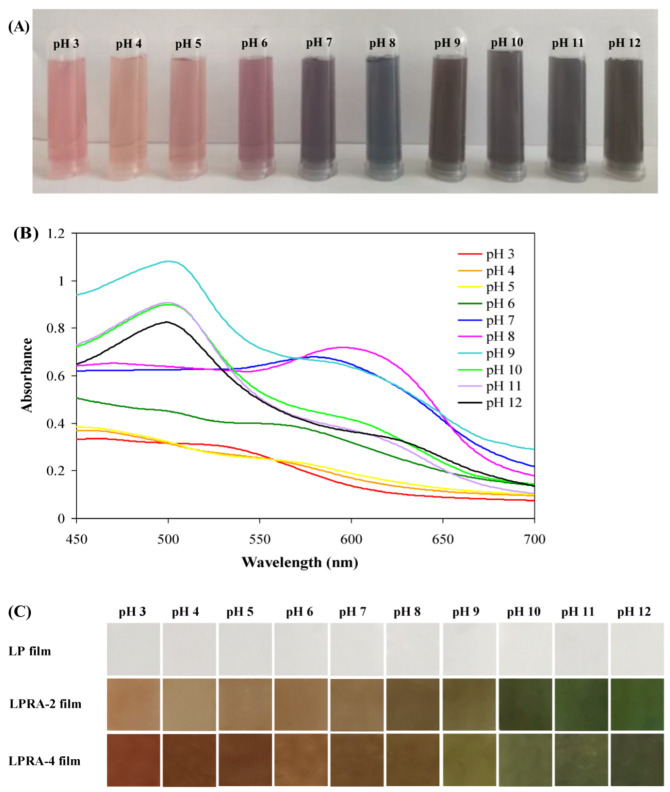

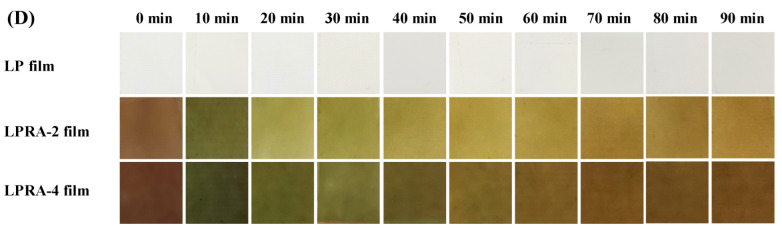
Color changes (**A**) and UV/Vis (**B**) spectra of RA in pH 3–12 buffers, and the color changes of LP film, LPRA-2 film, and LPRA-4 film in pH 3–12 buffers (**C**) and ammonia gas (**D**).

The color changes of the RA at different pH levels are shown in Figure 8A. The RA were pink at pH 3−6, greyish-purple at pH 7, blue at pH 8, and greyish-green at pH 9–12. The pH changes can also cause reversible structural transformations in anthocyanins, which has a dramatic influence on their colors [44]. The color changes of the anthocyanins at different pH levels were related to the structural transformation of the anthocyanins from flavonoid cations to anionic quinoids and chalcones at different acid–base conditions [45]. The UV/Vis spectra of the RA are shown in Figure 8B. The absorption bands of the RA gradually shifted from 530 nm (at pH 3) to 615 nm (at pH 8) and to 500 nm (at pH 12), confirming the structural changes of the anthocyanins at different pH levels. The pH sensitivity of the LP, LPRA-2, and LPRA-4 films is shown in Figure 8C. The LP film without RA was colorless at different pH levels. The LPRA-2 and LPRA-4 films were reddish brown at pH 3−7, olive at pH 8−9 and dark green at pH 10−12. The color changes of the films in ammonia gas are shown in Figure 8D. Under the action of ammonia gas, the LPRA-2 and LPRA-4 films changed from reddish brown to dark green, and finally brown. Other studies also reported that anthocyanin-rich films turned yellow in response to ammonia, which was related to the formation of chalcones under ammonia gas [24,46]. The color changes of the LPRA-2 and LPRA-4 films under ammonia gas happened because the volatile ammonia entered into the films and created an alkaline condition for the anthocyanins.

### 3.13. Application of Films

Shrimp is a protein-rich food that is highly perishable [47]. Due to possessing pH-sensitive and ammonia-sensitive properties, the LPRA-2 and LPRA-4 films were further used to indicate the freshness degree of shrimp. As shown in Table 2, the TVB-N value of the shrimp on the third day was 25.98 mg/100 g, which exceeded the freshness limit value of 20 mg/100 g for the Chinese standard GB 2733-2015, and indicated that the shrimp had spoiled on the third day. Notably, the color of the LPRA-2 and LPRA-4 films turned from originally being reddish-brown to dark green on the third day, which could be well distinguished by the naked eye. After the third day, the TVB-N value of the shrimp continuously increased, and the color of the LPRA-2 and LPRA-4 films gradually changed to brown. The results indicated that the LPRA-2 and LPRA-4 films could be used to indicate the freshness degree of shrimp. In previous studies, LP films containing *L. chinense* flower anthocyanins [24], pitaya betacyanins [31], and cockscomb flower betacyanins [26] were also able to indicate the freshness of shrimp.

## 4. Conclusions

Anthocyanins isolated from *R. simsii* were added to LBG/PVA matrices to prepare shrimp freshness indicating films. The hydrogen bonds between the RA and LBG/PVA matrices made the films become compact, which increased the light/water vapor/oxygen barrier abilities, the mechanical properties and the thermal stability of the films. Due to the presence of the anthocyanins, the antioxidant activity and pH-/ammonia-sensitive properties of the films were elevated. The barrier, mechanical, thermal and antioxidant properties of the films increased with the increase of the RA content from 2% to 4%. In general, the LPRA-4 film had higher light/water vapor/oxygen barrier ability, mechanical properties, thermal stability, and antioxidant activity than the LPRA-2 film. Despite this, both the LPRA-2 and LPRA-4 films were effective in indicating the freshness of shrimp during cold storage. In the future, LPRA-2 and LPRA-4 films can be used as smart packaging and antioxidant packaging materials in the food industry.

## Figures and Tables

**Table 1:** Appearance, thickness, and color values of LP film, LPRA-2 film, and LPRA-4 film.

Film	Appearance	Thickness (mm)	L*	a*	b*	Δ*E*
LP film	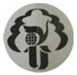	0.090 ± 0.003 ^a^	88.95 ± 0.06 ^a^	−0.71 ± 0.01 ^c^	0.54 ± 0.01 ^b^	1.65 ± 0.06 ^c^
LPRA-2 film	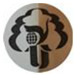	0.089 ± 0.004 ^a^	68.91 ± 0.87 ^b^	7.66 ± 0.53 ^b^	16.20 ± 1.07 ^a^	28.41 ± 1.44 ^b^
LPRA-4 film	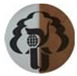	0.087 ± 0.002 ^b^	59.60 ± 1.08 ^c^	10.61 ± 0.57 ^a^	18.05 ± 0.34 ^a^	37.62 ± 1.22 ^a^

Values are given as mean ± standard deviation (n = 10 for film thickness, n = 3 for L*, a*, b*, and Δ*E*). Different letters in the same column indicate significant difference (*p* < 0.05). L*: lightness; a*: redness; b*: yellowness; Δ*E*: total color difference.

**Table 2:** The change of TVB-N levels in the shrimp and the color changes of LP film, LPRA-2 film, and LPRA-4 film.

Time (day)	TVB-N Level (mg/100 g)	LP film	LPRA-2 film	LPRA-4 film
0	5.10 ± 0.31 ^g^	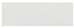	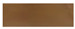	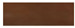
1	11.83 ± 0.25 ^f^	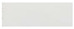	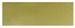	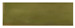
2	17.38 ± 0.12 ^e^	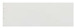	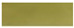	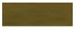
3	25.93 ± 0.57 ^d^	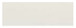	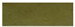	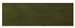
4	33.59 ± 0.13 ^c^	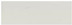	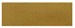	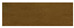
5	41.68 ± 0.23 ^b^	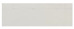	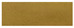	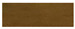
6	56.35 ± 0.19 ^a^	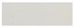	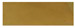	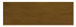

Values are given as mean ± standard deviation (n = 3 for TVB-N level). Different letters in the same column indicate significant difference (*p* < 0.05).

## Data Availability

The data presented in this study are available on request from the corresponding author.
